# Supplementary figures and images for: Neotaphonomic characteristics of vertebrate site formation in underwater caves
Source: PLoS One. 2026 Jul 15;21(7):e0343896. doi: 10.1371/journal.pone.0343896 (PMC13372153; doi:10.1371/journal.pone.0343896)

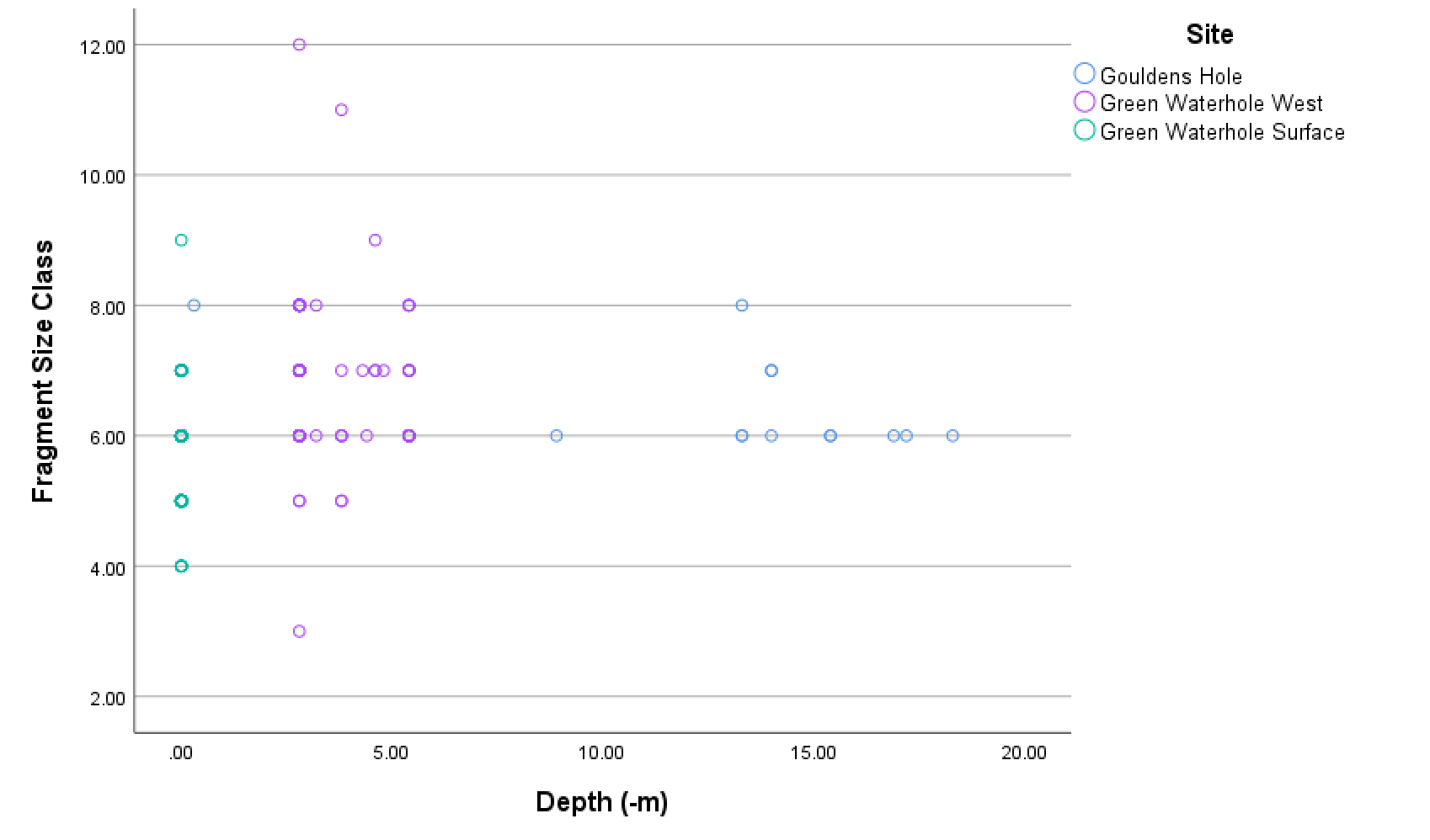

Supplement: S1 Fig — (TIF) [file pone.0343896.s003.tif]

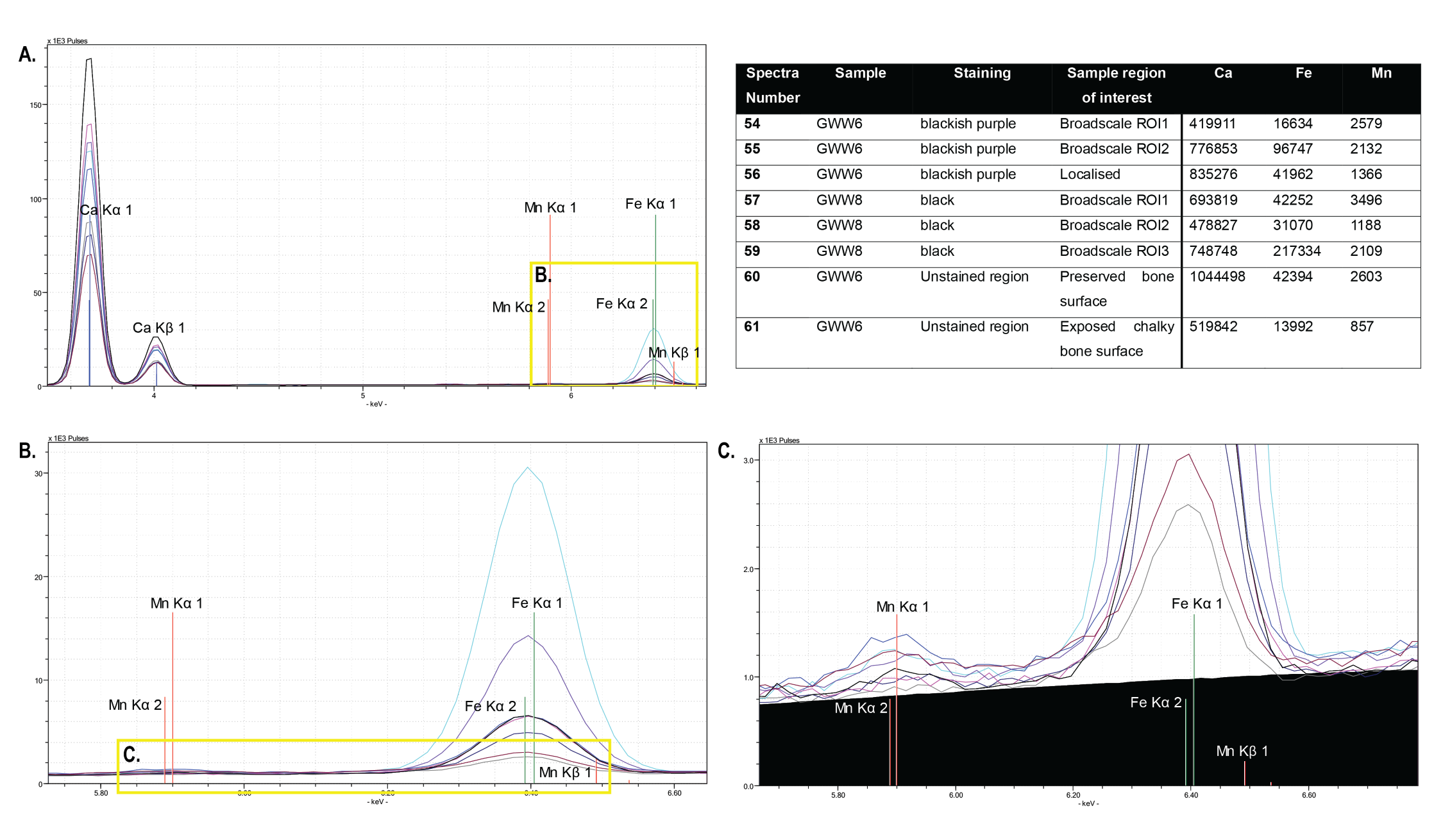

Supplement: S2 Fig — Associated spectral values and data provided in table. A: complete spectra; B: spectra highlighting Mn and Fe; C: focused view of Mn peaks. (TIF) [file pone.0343896.s004.tif]

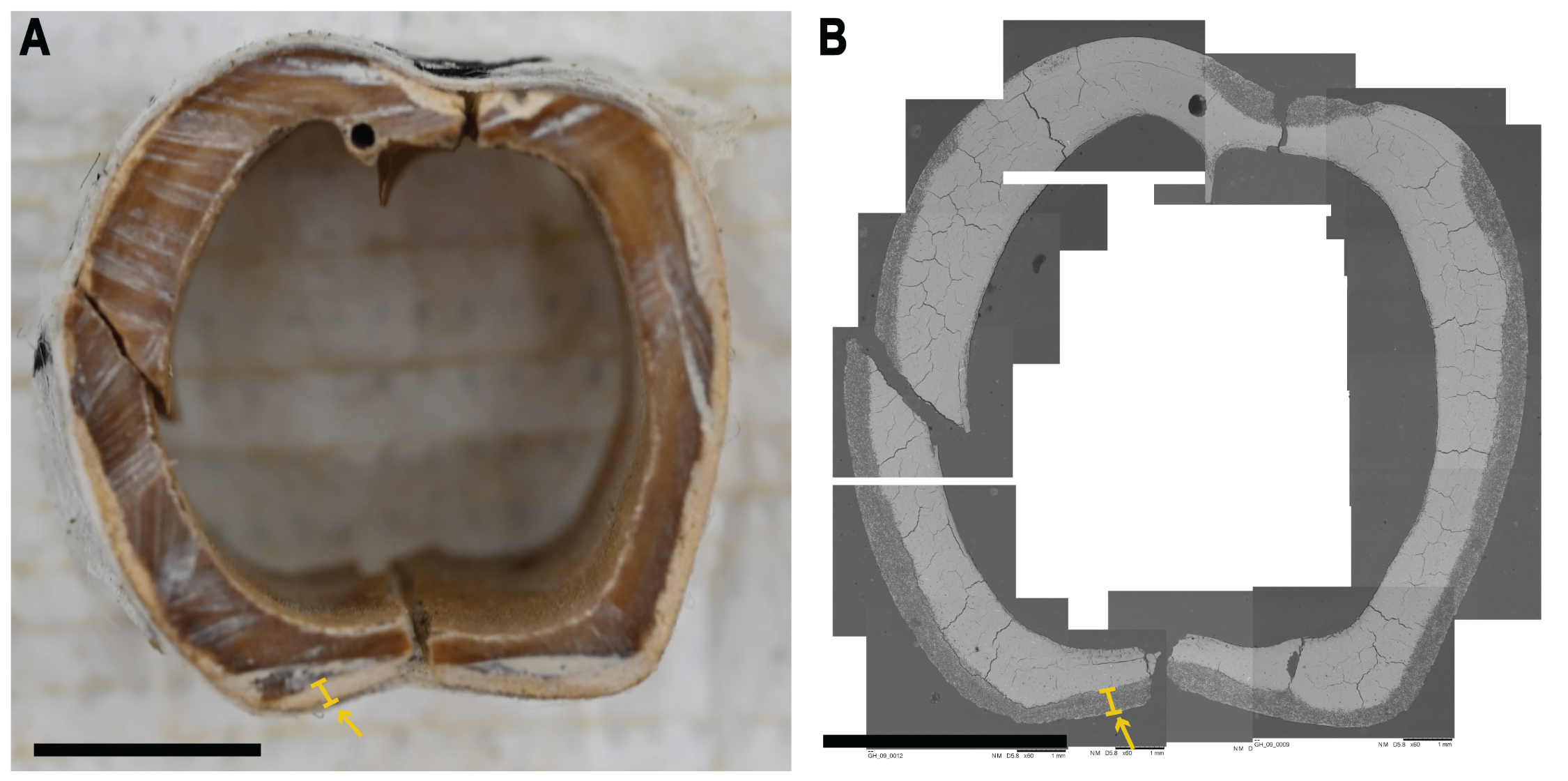

Supplement: S3 Fig — Yellow arrow and indicator identify the area that represents both a chalky degradation and tunnelling. Scale bars represent 5 mm. (TIF) [file pone.0343896.s005.tif]
